# Non-destructive Detection of Insect Foreign Bodies in Finishing Tea Product Based on Terahertz Spectrum and Image

**DOI:** 10.3389/fnut.2021.757491

**Published:** 2021-10-18

**Authors:** Xudong Sun, Jiajun Li, Yun Shen, Wenping Li

**Affiliations:** ^1^School of Mechatronics and Vehicle Engineering, East China Jiaotong University, Nanchang, China; ^2^Institute of Space Science and Technology, Nanchang University, Nanchang, China; ^3^School of Science, Nanchang University, Nanchang, China; ^4^Qingdao Quenda Terahertz Technology Co., Ltd, Qingdao, China

**Keywords:** terahertz, time-domain spectroscopy, time-resolved, imaging, foreign body

## Abstract

Non-destructive testing of low-density and organic foreign bodies is the main challenge for food safety control. Terahertz time-domain spectroscopy (THz-TDS) and imaging technologies were applied to explore the feasibility of detection for insect foreign bodies in the finishing tea products. THz-TDS of tea leaves and foreign bodies of insects demonstrated significant differences in terms of time domain and frequency signals in the range of 0.3–1.0 THz. These signals were corrected by the use of adaptive iteratively reweighted penalized least squares (AirPLS), asymmetric least squares (AsLS), and baseline estimation and de-noising using sparsity (BEADS) for reducing baseline drift and enhancing effective spectral information. The K-nearest neighbor (KNN) and partial least squares discrimination analysis (PLS-DA) models showed the best performance after AirPLS correction with the prediction accuracy of 98 and 100%, respectively. In addition, the locations and outlines of insect bodies could be clearly presented *via* the THz-TDS image. These results suggested that THz-TDS spectroscopy and imaging provide an alternative tool for the detection of insect foreign bodies in finishing tea products.

## Introduction

During processing, the food matrix is easy to be contaminated by foreign bodies, such as insects, metal, sand, plastics, hair, and so on, resulting in physical pollution (1). Foreign bodies are the biggest single source of customer complaints on many food manufacturers, retailers, and enforcement authorities (2). In addition, food foreign contamination issues will cause conflicts between manufacturers and consumers, and importers and exporters, which can only be reduced rather than eradicated. Therefore, non-destructive testing technologies used to scan and exclude foreign bodies will be one of the hot issues for future food analysis.

As a current mainstream technology, X-ray imaging is conducted based on the density difference between food matrix and foreign bodies. This method can be applied to detect metal and extended to high-density plastics. However, the low-density and organic foreign bodies are beyond their limitation (3), which poses big challenges for foreign bodies detection in food processing.

Terahertz wave locates between microwave and infrared light, with frequency ranging from 0.1 to 10.0 THz and wavelengths from 0.03 to 30 mm. Terahertz (THz) has low energy transmission, and the photon energy of THz wave at 1 THz stands at about 1 meV, which prevents from photo-ionization radiation damage to biological samples. With such low transmissivity, THz can penetrate through non-polar packaging materials like paper, and plastic, etc. to obtain the THz spectral and image information of the food matrix, serving as the optimal detection method for low-density and organic foreign bodies (4, 5).

Jördens and Koch (6) firstly adopted THz time domain spectroscopy (THz-TDS) technology to detect metal, sand, and stone foreign bodies imbedded in chocolate bars. They found that these foreign bodies resulted in the delay of the THz time-domain signal and the second wave peak appeared in the waveform, showcasing the potential advantage of THz-TDS technology for detecting foreign bodies in the food matrix. THz-TDS technology can not only reflect the variation of amplitude and phase that indicate the differences of food substrate and foreign bodies (7, 8) but also identify low-density foreign bodies like insect bodies imbedded in food substrate. Shin et al. (7) detected food substrates and insect foreign bodies by means of THz-TDS technology and found that different food substrates and insects showed varied absorption coefficients and refractive indexes. From the THz reflection image, insect bodies could be observed from the food substrate. Therefore, it showed that the THz imaging technology based on complex refractive index mapping was a helpful tool for food quality assessment (9). Shen et al. (10) discriminated wheat and impurities through THz spectral imaging technology combined with a convolutional neural network (CNN). They found that wheat and foreign bodies had shown differences in time domain signal, absorption coefficient, and refractive index, with the detection accuracy reaching 97.86%. Jiang et al. (11) identified foreign bodies of metals, stones, glass fragments, and wood in different depths of cereal by using THz reflection imaging technology. They found that the THz reflected signals of the cereal and foreign bodies showed significant differences. When foreign bodies were imbedded, the medium of food substrate was significantly changed. On the verge of medium change, the THz-TDS spectra can easily be subject to scattering that further leads to baseline drift. Therefore, the application of baseline correction algorithms can improve the detection accuracy of THz-TDS technology (12, 13). In addition, the time delay of the THz-TDS signal, affected by the change of medium, has remarkable time-resolved characteristics.

As continuous THz waves can reflect the amplitude changes of food substrate and foreign bodies, it has been adopted by researchers to detect foreign bodies. OK et al. (4, 14) developed a high-speed THz imaging system on the basis of continuous wave (CW) THz imaging modal, which can simultaneously realize a fast scanning speed of 80 mm/s, wide range detection of about 150 mm, and high resolution of 2.83 mm at 210 GHz. Yu et al. (15) developed a THz imaging system operating at 0.3 THz by means of an array sensor structure, which can be used to detect foreign bodies in food conveyed at a speed higher than 20 m/min. The research mentioned above serves as powerful evidence that CW THz scanning imaging enjoys great potential in detecting foreign bodies in food on the assembly lines.

The objective of this study is to study the response characteristics of spectra and images at the THz frequency band of food substrate and foreign bodies in a bid to find out the optimal detection method for low-density and organic foreign bodies. What has been done is presented as follows: first, the response characteristics of THz-TDS of tea substrate and insect bodies were analyzed and the corresponding model was established. Second, the response characteristics of THz time-resolved images of tea substrate and insect bodies were studied.

## Materials and Methods

### Experimental Samples

Pests feed on tea leaves during the growth stage and are easily mixed into the raw materials, forming foreign bodies pollution. Insect body pollution accounted for 25% (1) among all foreign bodies, so barley pests and beetles were selected as the foreign bodies. The insects and Longjing Green tea were collected from the local market.

The samples were prepared according to the following steps. First, the samples of tea leaves and insects were cleaned before being put into the drying baker with a controlled temperature of 50°C for 12 h. Second, the dried tea leaves were grounded into powder and screened out by a 200-mesh sieve. Third, the powder was pressed at a stress of 5,000 kg/cm^2^ by a tableting machine (HY-12, Tianjin Tianguang Optical Instrument Co., LTD, Tianjin, China) for 3 min so that the insect bodies could be buried into the tea powder. Considering that the insect foreign bodies in tea were mostly fragments, the legs and shells of the insects were directly pressed into the tablet. Lastly, tea powder and insect bodies were made into one tablet with a diameter of 13 mm and the thickness varied between 0.95 and 1.10 mm. The discrimination of THz-TDS spectroscopy generally required the appropriate quantity of samples for building trusted models. Therefore, this experiment was conducted twice with the interval of 1 week for two populations, the first (*n* = 125) for modeling and the last for prediction (*n* = 51).

For studying the changes of the pattern in the THz-TDS image of tea mixed with insect foreign material, the barley pest was imbedded into the bottom of tea leaves with a thickness of about 2 mm. To avoid the influence of moisture content, both tea leaves and barley pest samples were controlled by drying them at 50°C for 12 h.

### THz-TDS Spectroscopy System

The THz-TDS system was constituted by a femtosecond laser device, a photoconductive antenna, a time delay device, and a data collection and processing software (TDS1008, BATOP Optoelectronics, Gena, Germany). The femtosecond laser with a wavelength of 780 nm generates femtosecond pulse which is split by the spectroscope into two light beams, one of which is pump light with a power of 100 mW and the other is a probe light. The pump light then generates a THz pulse after passing through the GaAs photoconductive antenna. Through the focusing lens, the THz pulse reaches the sample which carries the sample information reaching the detector. At the same time, another beam of probe light reaches the detector crystal after passing the delayer so that the detector transforms the received signal into an electrical signal to the computer, thus obtaining the THz spectral information of the samples. During the experiment, the THz frequency was 0.05 to 4 THz and the temperature was 24°C. Before the samples were placed into the experiment module, the devices were preheated for 30 min after being fed with dry nitrogen. Two spectra were collected for one sample and averaged for calibration and prediction.

The original THz signal is a time domain spectrum that needs to be transformed into THz frequency domain spectrum by means of fast Fourier transform (FFT). The formula is shown as follows (16).


(1) $$\tilde{E}\left(\omega \right)=A\left(\omega \right)e^{-i\phi \left(\omega \right)}=\int dtE\left(t\right)e^{-i\omega t}$$

where *A(*ω*)* is the amplitude of the electric field, ϕ*(*ω*)* is the phase position of the time-domain spectrum, and *E(t)* is the time-domain spectrum.

### THz-TDS Imaging System

**Figure 1:**
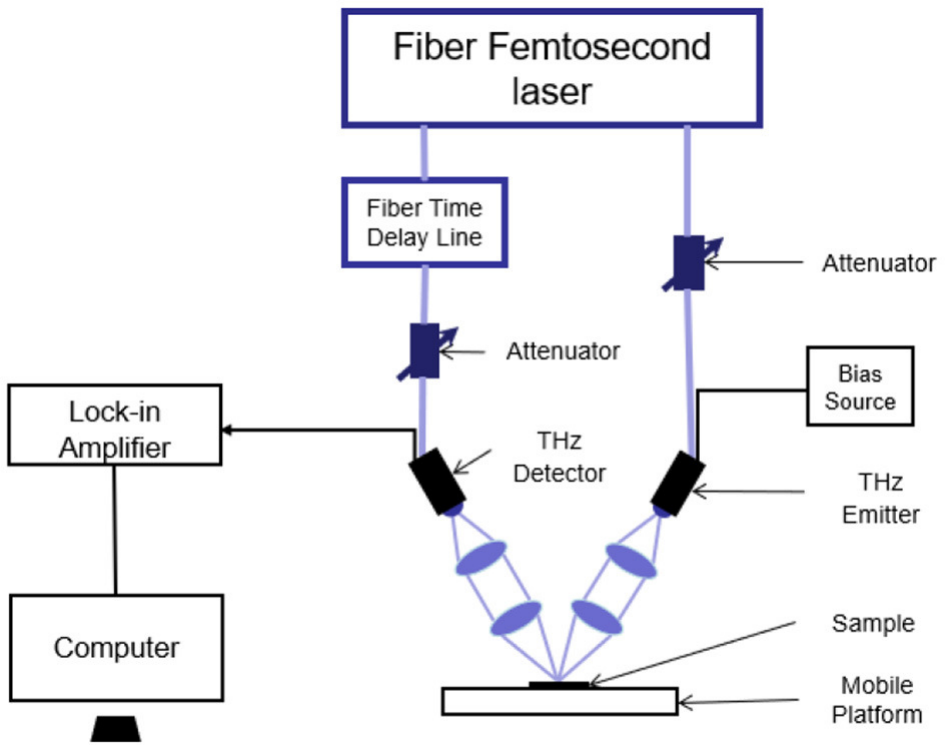
Semantic diagram of optical path in QT-TO100 terahertz (THz) device.

The THz image scanning system is a three-dimensional imaging system (QT-TO1000, Terahertz Technology Co., Ltd, Qingdao, China) (Figure 1). The femtosecond laser generates femtosecond pulses which are divided into pump light and probe light. The pump light enters the THz emitter and generates THz radiation. The THz pulse emitted by the emitter reaches the samples after being focused. Then the THz reflection wave carrying sample information is focused on the detector when the probe light also gets to the detector crystal. During the experiment, the THz frequency was 0.1 to 3.5 THz with the maximum scanning area of 100 × 100 mm, maximum detection thickness of 9 mm, and maximum imaging speed of 60 pixels/s.

The room temperature and the humidity were set at 26.5°C and 65%, respectively. After half an hour of preheating, the devices were used to collect THz reflection images. The samples were placed on the moving platform whose height was then adjusted to get the optimal THz reflection signals. With metal as the reflection floor, the images were scanned in a dot-to-dot mode. The X-Y platform moved with the step of 0.2 mm. Images were collected under the control of software and the THz three-dimensional tomographic images.

### Data Processing

#### AsLS Correction

As an alternative and general method for baseline estimation, asymmetric least squares (AsLS) was proposed by Eilers et al. (17). AsLS is only correlated to parametric time distortion, so it does not require prior information of waveform or baseline. For AsLS, Whittaker smoother is used to obtain baseline estimation with slow change. The valid baseline estimators can be obtained by combining a smoother with an asymmetric weighting of the smoothed trend deviation. By means of the least square penalty function, the noises can be removed and baseline trends can be kept balanced. The relationship between estimated baseline (z) and spectral signal (y) can be expressed as follows:


(2) $$\begin{array}{l} z={\left(W+\lambda D^{T}D\right)}^{-1}Wy \end{array}$$

(3) $$\omega_{t}=\{\begin{array}{cc} p, & y_{i}\geq z_{i}\\ 1-p & y_{i}\geq z_{i} \end{array}$$

where ***W*** is the diagonal matrix whose diagonal element is **ω** (also serves as the weight vector); ***p*** is an asymmetrical parameter, often set at 0.001–0.1, and is used to adjust the balance between the fitness and smoothness of baseline; ***D*** is second order difference matrix. With certain values of λ and ***p***, the smooth baseline undergoes iterations until ω gets stabilized or the number of iteration reaches the predefined number.

#### Adaptive Iteratively Reweighted Penalized Least Squares (AirPLS) Correction

Adaptive iteratively reweighted penalized least squares is a rapid and flexible baseline fitting algorithm based on penalized least square method, in which irregular and complicated baselines can be drawn near by means of iteratively re-weighted least squares. During iteration, the weights of the residual sum of squares (RSS) between the fitting baseline and the original signal are adaptively adjusted in an iterative process. Based on an adaptive iterative reweighting process similar to AsLS, AirPLS supplements a penalized parameter to control the smoothness of fitting baselines. Through different methods, AirPLS can acquire updated weightings in a self-adopted manner. The new **ω_*i*_** is expressed as follows (18):


(4) $$\omega_{i}=\{\begin{array}{cc} 0, & y_{i}\geq z_{i}\\ e^{\frac{t\left(y_{i}-z_{i}\right)}{\left|d\right|}}, & y_{i}\leq z_{i} \end{array}$$

where ***t*** is the number of iteration and ***d*** is the controls parameter. If the value of signal ***y***_***i***_ is larger than that of baseline ***z***_i_, then signal ***y***_***i***_ is a part of the absorption peak, with a weight set at *0*; otherwise, the weight of signal ***y***_***i***_ is updated in accordance with Formula (4). When ***t*** reaches the maximum number of iteration or |*d*| <0.001 × |*y*|, the iteration ends.

#### BEADS Correction

Baseline estimation and de-noising using sparsity, proposed by Ning et al. (19), is a method based on modeling a series of spectral peaks as sparse signals with sparse derivative, which performs the complete decomposition of spectrum in net signal, baseline, and noise. The spectral baseline and noise are modeled as low-pass signal and high-pass component, respectively, while the analysis peak is described as a sparse signal. An asymmetrical penalty function is used to demonstrate the positivity of the spectral peak. The baseline estimation and de-noising using sparsity (BEADS) method for minimized asymmetrical penalized function is defined as follows (12).

Step one: initialization of parameters. In the formula $d=B^{T}BA^{-1}y-\lambda_{0}A^{-1}\left(1-r\right)/2$, ***x*** is substituted for ***y***. ***y*** is the original spectrum and ***y*** equals ***x*** plus ***f*** plus ***w*** (*y* = *x* + *f* +*w*). ***x*** is the sparse signal, ***f*** is the spectral baseline and ***w*** is the spectral noise. *A* and *B* are both banded convolution matrices. The high pass filtering *H* is defined as *H* = *BA*^−1^, with two parameters of an order and cut-off frequency. **λ** is a regularization parameter that controls the sparsity of peaks while ***r*** is a parameter with a value above *0*.

Step two: repeat the following calculations until convergence.


(5) $$\Psi =\{\begin{array}{c} (1+r)/(4|v|),\left|v\right|\geq \left|\delta \right|\\ (1+r)/(4\left|\delta \right|),\left|v\right|\leq \left|\delta \right| \end{array}$$

(6) $$\begin{array}{l} \Lambda_{i}=\frac{\varphi^{\prime }\left(\left[D_{i}x\right]\right)}{\left[D_{i}x\right]},i=0,\ldots P \end{array}$$

(7) $$\begin{array}{l} M=2\lambda_{0}\text{Ψ}+\overset{p}{\underset{i=1}{\sum }}\lambda_{i}D_{i}^{T}\Lambda_{i}D_{i} \end{array}$$

(8) $$\begin{array}{l} x=A{\left(B^{T}B+A^{T}MA\right)}^{-1}d \end{array}$$

where **δ** is a parameter that is adjusted to maintain the effective sparsity enhancement of the original non-differentiable penalty function and avoid the value issues appearing in the MM optimized algorithm. ***P*** is the order of derivative and the value of ***i*** can be set from ***0*** to ***P***. ***D***_***i***_ is the ***i*** order difference matrix and **ψ** is the diagonal matrix. **Λ** is a diagonal matrix with the diagonal element $\Lambda \left(\nu \right){=}^{\phi \left(\nu \right)}{/}_{\nu }$ in which ***v*** is a scalar quantity. ***M*** is banded matrix and the final baseline estimation can be expressed as *f* = *y*−*x*−*BA*^−1^ *(y – x)*.

### Modeling Algorithm

#### KNN Algorithm

K-nearest neighbor is a classification method without definitive training stages (20). When the input is classified, ***k*** nearest neighbors are searched in the training data through a given distance measure. If the majority of these ***k*** nearest neighbors fall into a certain category, then the input can also be classified into this category. The distance measured herein is the Euclidean distance.

#### PLS-DA Algorithm

Partial least squares discrimination analysis is a supervised mode of partial least squares analysis. That is to say, in the process of data analysis, the information of grouped samples is already known so that the characteristic variable of each group can be better selected and distinguished to determine the relations between samples. The principle is to establish a regression model based on partial least squares (PLS) regression while reducing the dimensionality of the data and to judge the regression results. Among them, PLS mainly establishes a linear model through multiple linear regressions. Discrimination analysis (DA) refers to the discriminant analysis of the regression results.

## Results and Discussion

### Characteristics of THz Spectra

**Figure 2:**
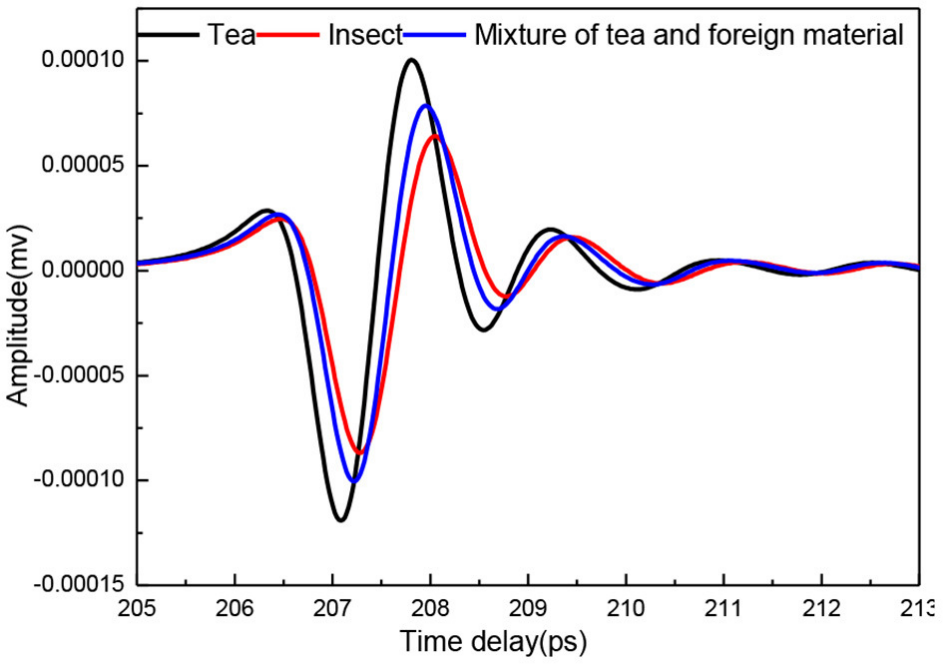
THz time-domain spectra of tea, insect, and their mixture tablets.

Figure 2 shows the mean THz time-domain spectra of pure tea-leaf powder tablet, pure insect powder tablet, and their mixture tablet of 25% insect foreign material. It could be observed that the spectrum of the pure insect sample had lower intensity than that of the pure tea sample, and the spectral intensity of the sample with insect foreign material was between the pure tea and the pure insect. This indicated that the insect foreign material showed greater absorption to THz radiation. The insect contained fat, protein, and other compounds which had strong absorption of THz radiation. Meanwhile, there were some differences in the time delay of the THz signal between the insect and the tea samples.

**Figure 3:**
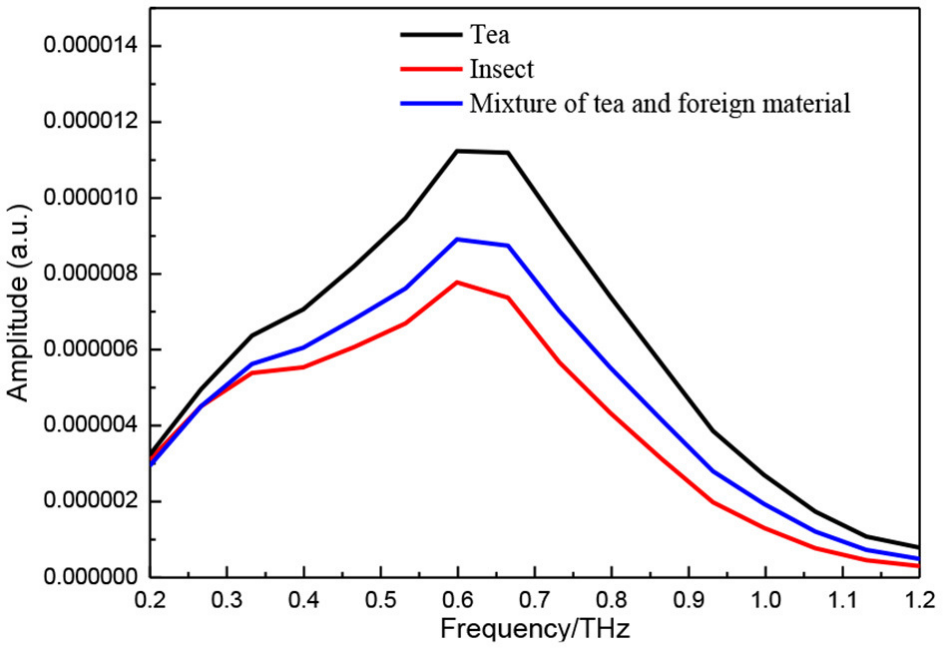
Frequency-domain spectra of tea, insect, and their mixture tablets.

The average terahertz frequency domain spectra calculated with FFT are shown in Figure 3. The spectral shapes of tea and insect material at 0.3 to 1.2 THz differed from each other, with the spectrum of the insect material sample, basically, lower than that of the pure tea sample. In the frequency of 0.2–0.3 THz, the spectral of the samples showed a slight difference, and the samples with insect foreign material were more similar to those of the pure insects. In the frequency of 0.3–1.2 THz, the spectrum of the insect material sample was obviously lower than that of the tea material sample, and the tea sample with insects was higher than that of pure insect and lower than that of pure tea. This may be caused by the insect material contained in tea which presented different absorption and scattering at the different THz frequencies.

### THz Spectral Baseline Correction

**Figure 4:**
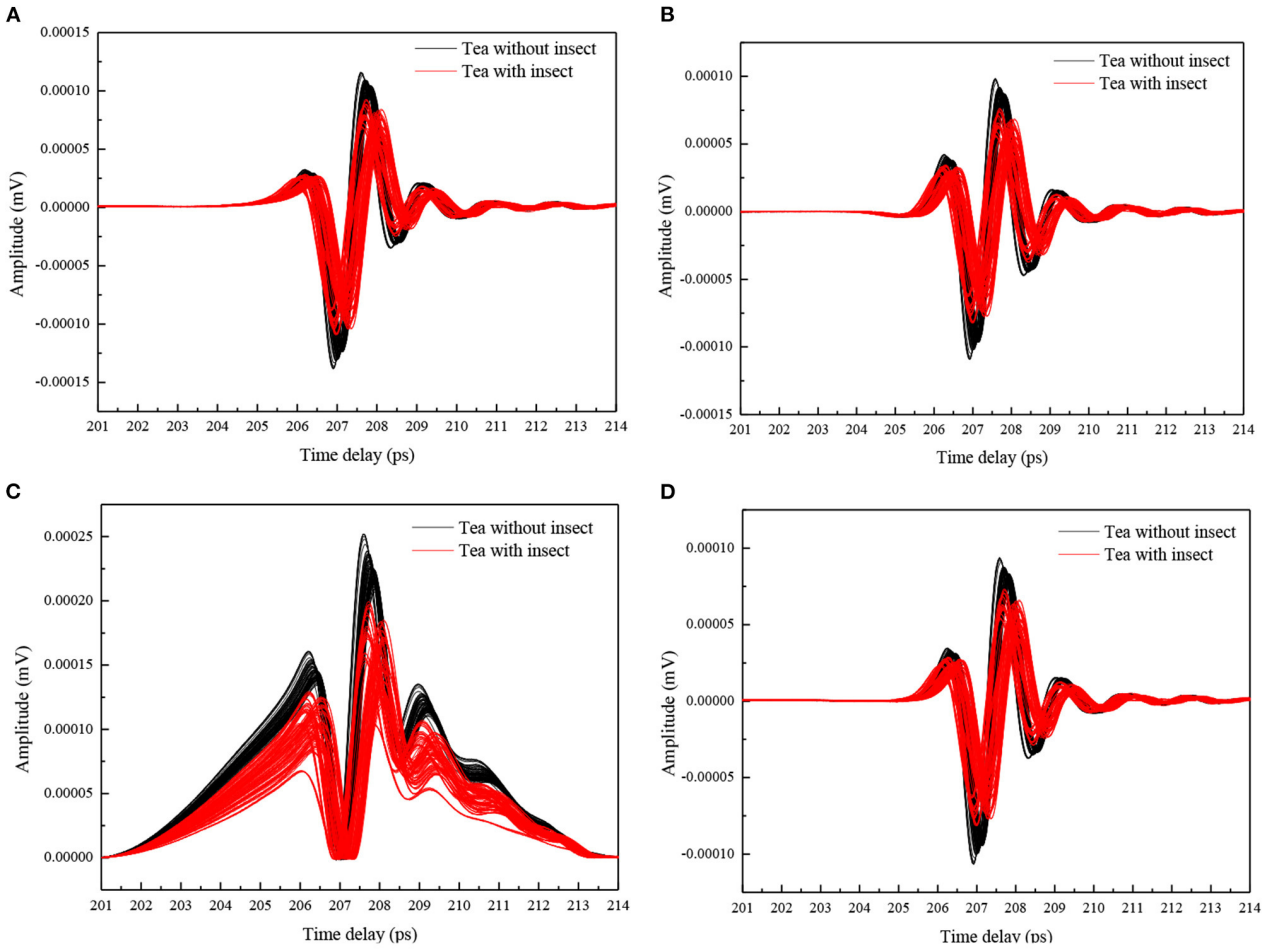
Raw spectra **(A)** and the spectra after baseline correction with AsLS **(B)**, AirPLS **(C)**, and BEADS **(D)**.

The reflection and transmission characteristics of THz radiation will be changed when the tea powder is contaminated by the foreign bodies of insects, and these properties can be applied to identify the foreign bodies (6). However, the scatter effects are easily caused because of particle size and shape of tea leaves and it cannot be ignored for foreign bodies identification using THz-TDS spectroscopy. The correction algorithms of AsLS, AirPLS, and BEADS were adopted for reducing baseline shift, the spectra before and after correction are shown in Figure 4. The correct recognition rates were increased from 90 to 98% and 100% for K-nearest neighbor (KNN) and PLS-DA models, respectively. This indicated that the baseline correction could improve the accuracy of qualitative discrimination for THz-TDS spectroscopy. These correction methods also improved the accuracy of quantitative analysis of 2, 4-dichlorophenoxyacetic acid in the previous report (12).

### THz Spectral Calibration and Prediction

Table 1 compares the discriminative performance of baseline correction methods combined with different modeling methods and correspondingly summarized the accuracy results obtained from calibration and prediction sets. A detailed comparison based on the accuracy suggested that the best discriminant accuracy was obtained by using the KNN method combined with the AirPLS baseline correction method, with the discrimination accuracy in calibration and prediction sets reaching 100 and 98%, respectively. For models using the same chemometric method, those that underwent baseline correction showed better effect than those without. This may result from the fact that when the medium is changed, the THz time domain signals in original data are subject to scattering which leads to the baseline drift of THz spectral signals and destabilizes the model. As for baseline correction methods, the modeling set accuracy and prediction set accuracy of the AirPLS-KNN model and AirPLS-PLS-DA model reached 100 and 98%, and 93 and 100%, respectively, which were superior to those of BEADS and AsLS methods.

**Table 1:** Detection results of KNN and PLS-DA models of tea leaves with insects.

**Modeling method**	**Correction method**	**No. of misclassified samples in calibration set**	**Accuracy in calibration set (%)**	**No. of misclassified samples in prediction set**	**Accuracy in prediction (%)**
KNN	No	0	100.00	5	90.20
	AirPLS	0	100.00	1	98.04
	AsLS	0	100.00	4	92.16
	BEADS	0	100.00	4	92.16
PLS-DA	No	19	84.80	5	90.20
	AirPLS	9	92.80	0	100.00
	AsLS	14	88.80	0	100.00
	BEADS	13	89.60	0	100.00

The result in the current study was better than our previous reports in THz-TDS detection of insect foreign bodies in Green tea with an accuracy of 89% (21). The accuracy of 89% was close to this report of 90% when without scatter effect correction was applied. Our experiment was also slightly better than the THz-TDS application for wheat defect detection with an accuracy of 96% (22). The scatter effect is a common problem in THz-TDS measurement of solid state samples, especially for irregular granular samples (23). To reduce or remove baseline shifts, mathematical algorithms can be considered.

### THz-TDS Image Recognition

#### THz-TDS Reflection Image

**Figure 5:**
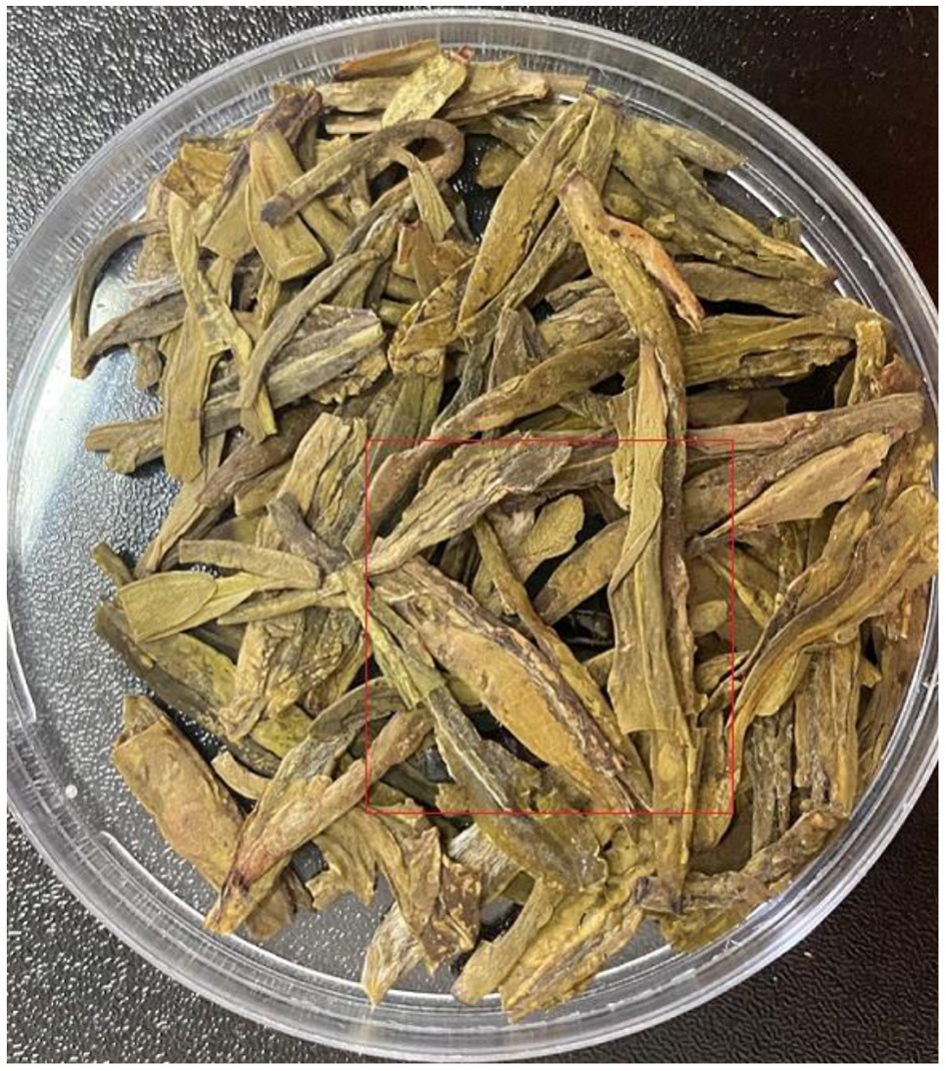
RGB photo of the sample for THz image scan with insect foreign material embedded into the bottom of tea products.

**Figure 6:**
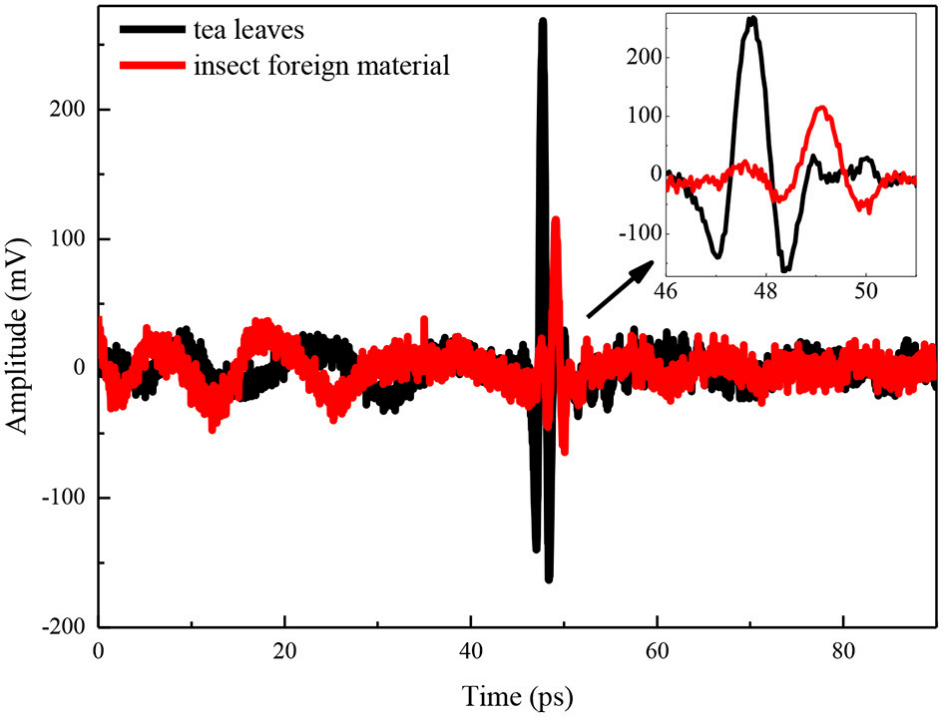
Time domain waveforms for the region of interest of tea leaves and insect foreign material.

**Figure 7:**
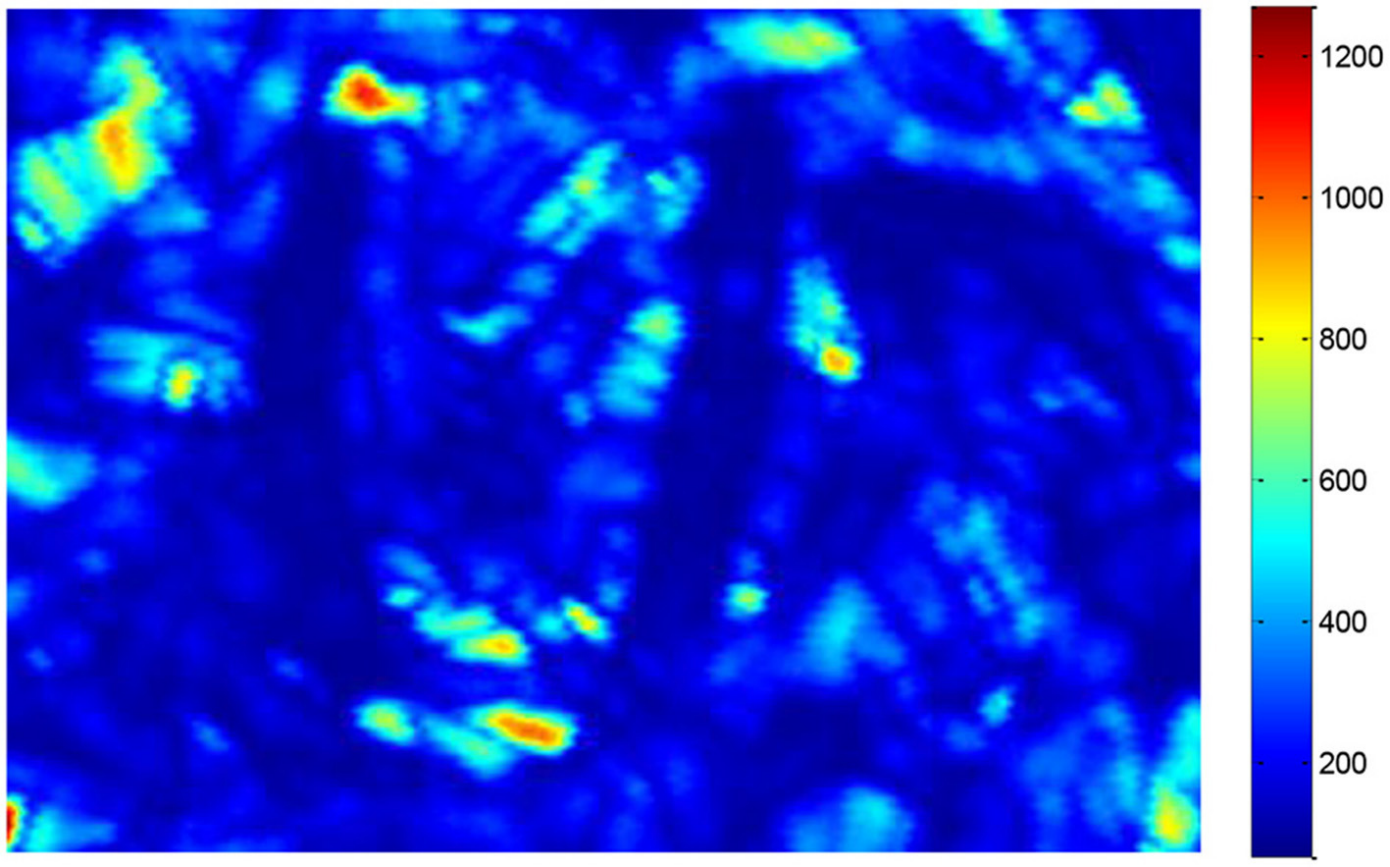
False color image obtained by THz reflection imaging (2,480 × 3,509 pixels) with grayscale values varying from minimum and maximum values (68-1272).

**Figure 8:**
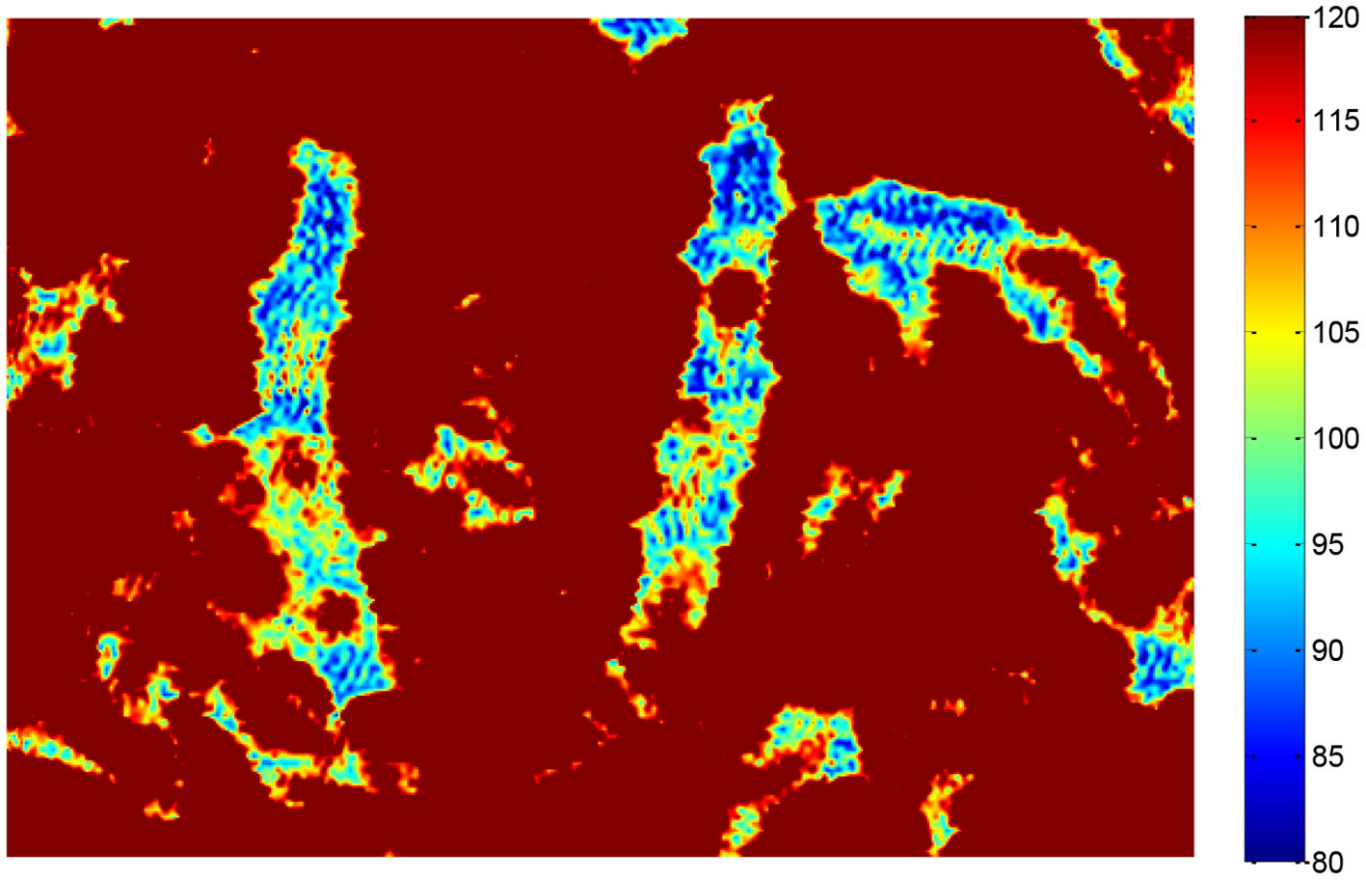
False color image for THz reflection imaging (2,480 × 3,509 pixels) with grayscale values adjusted from 80 to 120.

Terahertz time-domain spectroscopy imaging provided a potential tool for food foreign material detection, e.g., Jördens and Koch's report in the detection of metallic and non-metallic foreign bodies in chocolate by THz-TDS imaging (6). However, foreign bodies detection for tea products is more difficult than uniform solid samples because the THz wave is influenced by the status of randomly scattered tea leaves. We implanted two insect foreign bodies into the bottom of the tea leaves with a thickness of about 2 mm and put them into a petri dish with a diameter of 60 mm and a depth of 8 mm (Figure 5). The circular regions of interest for insects and tea leaves about 100 pixels were selected, and then the time domain waveforms were extracted from the two regions (Figure 6). The time delay could be observed from Figure 6 because the flight time of the THz wave at the insect location was longer than the region of tea leaves. The 2D THz-TDS image (2,480 × 3,509 pixels) was scanned with the movement step of 0.2 mm, and the minimum and maximum grayscale values fluctuated from 68 to 1,272 (Figure 7). When the grayscale values for visualization were limited to 80–120, the appearance of two worms could be found (Figure 8). Although several suspicious areas appeared, the insect foreign bodies could be judged by image processing algorithms combined with shape features. A good performance image could be obtained, e.g., THz-TDS images for the tablet of the walnut kernel and shell (24), but the scattering effect caused by non-homogeneous media was still a challenge for THz-TDS imaging practical application (25).

Both resolution and imaging speed should be considered for THz-TDS imaging applications. Our current study with an imaging time of 6 min/frame over a range of 35 × 25 mm was superior to a 140 GHz sub-wavelength imaging system of 2 h/frame for 60 × 60 mm (26). However, it was slightly lower than the sub-terahertz imaging system of 45 min/frame with a range of 80 × 60 mm (25). For the resolution, the sub-terahertz imaging system provided good contrast when it focuses on the homogeneous samples, e.g., mealworm in the chocolate bar (26) and crickets in noodle flour (25). A clear image was also obtained for non-homogeneous samples of tea leaves *via* controlling the flight time of the THz wave (Figure 7). Up to now, most of the publications did not involve model development and prediction and still remained in feasibility investigation. One of the priorities of future work should focus on image processing models and predictions. For example, using a large amount of data and models to evaluate the performance of THz-TDS imaging (10).

## Conclusions

Terahertz time-domain spectroscopy and imaging were applied to investigate the feasibility of non-destructive testing of insect foreign matters in finishing tea products. Results showed that the tea leaves and insect foreign bodies showed a significant difference in THz frequency based on THz-TDS signals and THz absorption coefficient. Baseline correction algorithms could be used to enhance the difference between tea leaves and insects bodies. The KNN and PLS-DA models showed the best result using AirPLS correction, with the prediction accuracy reaching 98 and 100%, respectively. These results indicated that THz-TDS could be adopted to detect insects' foreign bodies imbedded in tea leaves. In addition, the THz-TDS imaging provided a visual detection method for tea inclusion foreign bodies, and the position and the profile of the insect foreign bodies could be observed by using THz-TDS imaging.

## Data Availability Statement

The original contributions presented in the study are included in the article/supplementary material, further inquiries can be directed to the corresponding author/s.

## Author Contributions

XS: conceptualization, methodology, review and editing, project administration, funding acquisition, supervision, and resources. JL: spectra collection, formal analysis, and original draft. YS: THz-TDS system and supervision. WL: image collection. All authors contributed to the article and approved the submitted version.

## Funding

This work was supported by the National Natural Science Foundation of China (No. 31960497) and the Natural Science Foundation of Jiangxi Province (No. 20202BAB205009).

## Conflict of Interest

WL is employed by Qingdao Quenda Terahertz Technology Co., Ltd. The remaining authors declare that the research was conducted in the absence of any commercial or financial relationships that could be construed as a potential conflict of interest.

## Publisher's Note

All claims expressed in this article are solely those of the authors and do not necessarily represent those of their affiliated organizations, or those of the publisher, the editors and the reviewers. Any product that may be evaluated in this article, or claim that may be made by its manufacturer, is not guaranteed or endorsed by the publisher.
